# Supplementary material for: Efficient ethylene purification by a robust ethane-trapping porous organic cage
Source: Nat Commun. 2021 Jun 17;12:3703. doi: 10.1038/s41467-021-24042-7 (PMC8211788; doi:10.1038/s41467-021-24042-7)
Supplement: Supplementary file 1 — Supplementary Information [file 41467_2021_24042_MOESM1_ESM.pdf]

Supplementary Information

Efficient ethylene purification by a robust ethane-trapping porous organic cage

Kongzhao Su[1,2,4], Wenjing Wang[1,4], Shunfu Du[1,3], Chunqing Ji[1,2] & Daqiang Yuan[1,2,*]

[1]State Key Laboratory of Structure Chemistry, Fujian Institute of Research on the Structure of Matter, Chinese Academy of Sciences, Fuzhou, 350002 Fujian, China

[2]University of the Chinese Academy of Sciences, Beijing, 100049, China

[3]College of Chemistry, Fuzhou University, Fuzhou, 350116, China

[4]These authors contributed equally

Corresponding author. *E-mail address*: ydq@fjirsm.ac.cn (D.Q. Yuan).

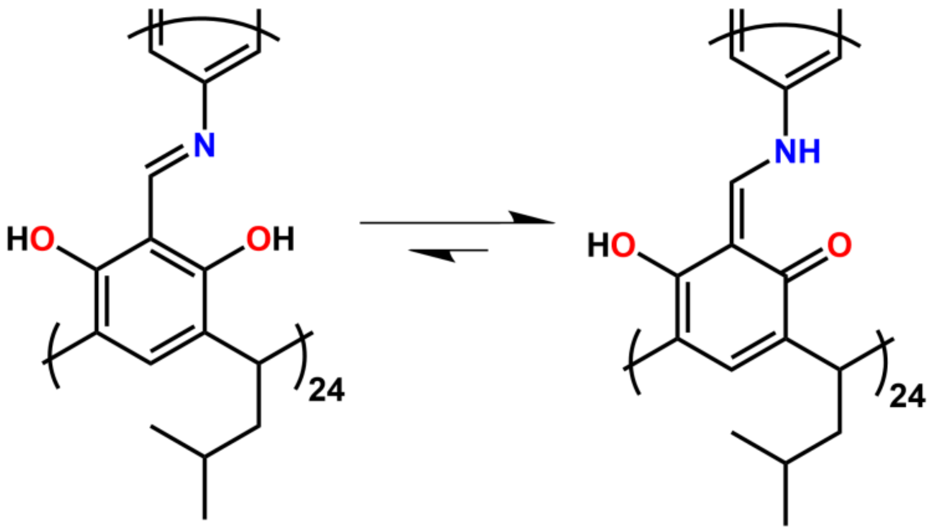

Supplementary Fig. 1 Keto–enol tautomerization within CPOC-301.

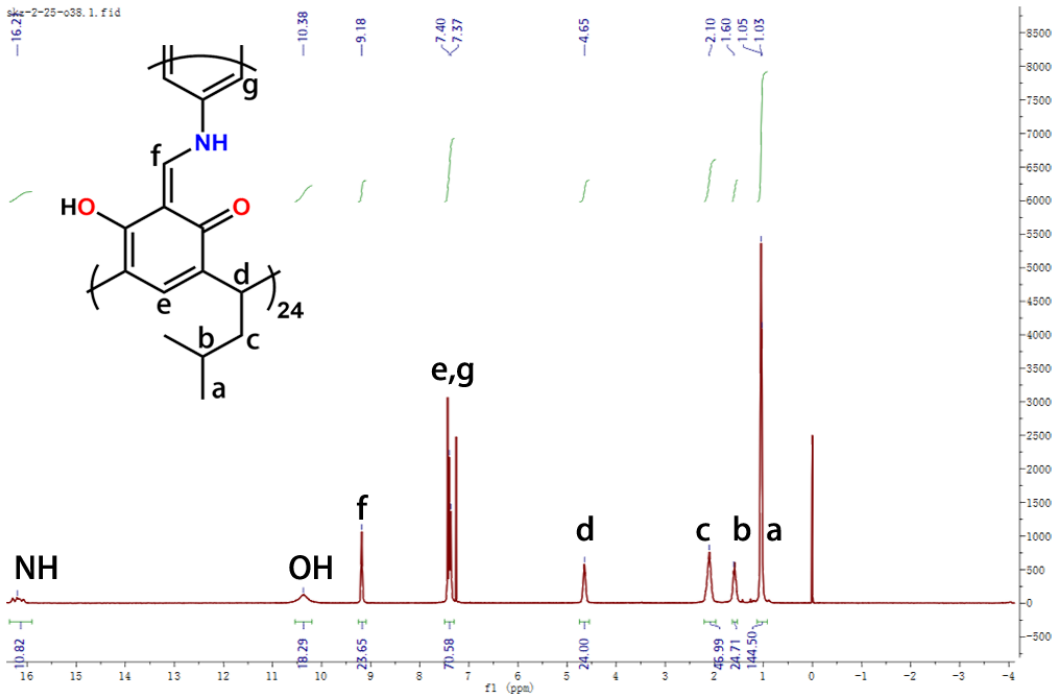

Supplementary Fig. 2 ^{1}HNMR of CPOC-301 ($CDCl_3$, 400 MHz, 298 K). The lower integral areas at 10.38 and 16.21 ppm are due to the active hydrogen atoms.

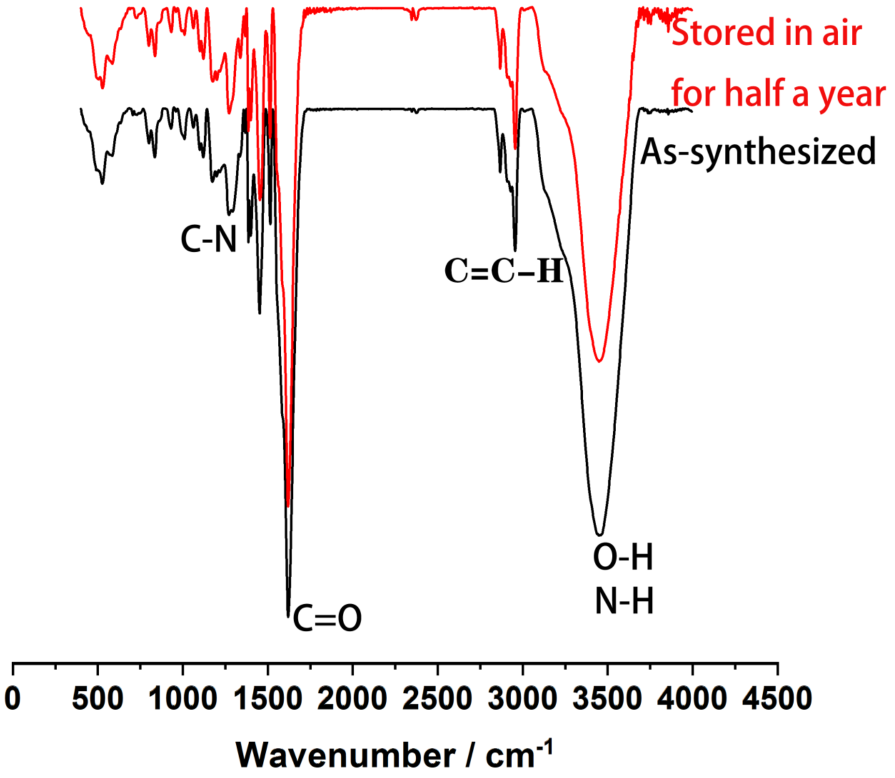

Supplementary Fig. 3 FT-IR spectra of CPOC-301.

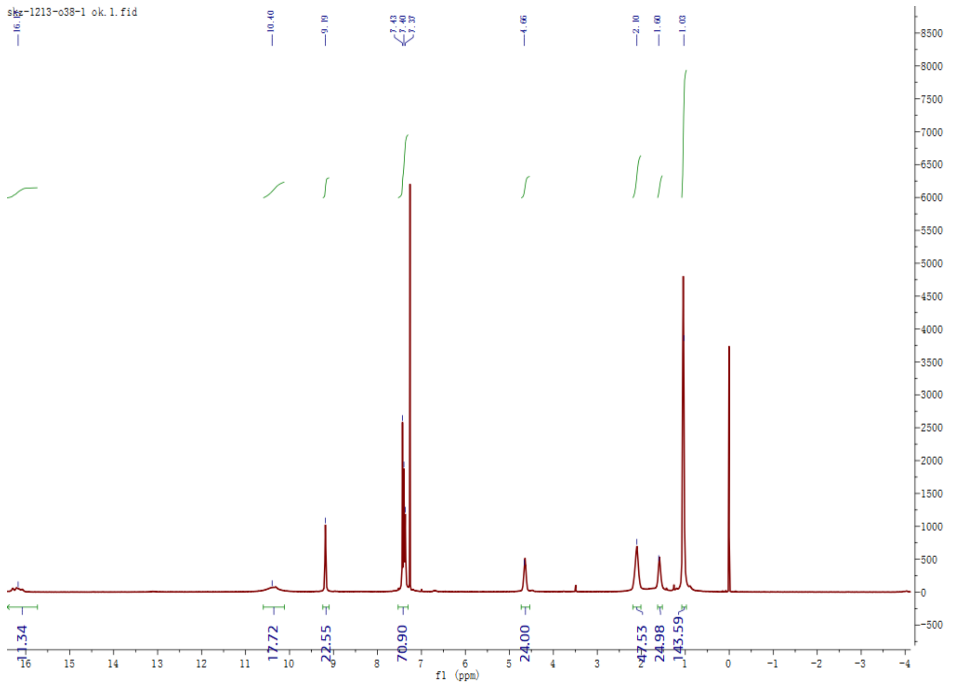

Supplementary Fig. 4 ^{1}HNMR of CPOC-301 after storing in air for half a year ($CDCl_3$, 400 MHz, 298 K). The lower integral areas at 10.40 and 16.19 ppm are due to the active hydrogen atoms.

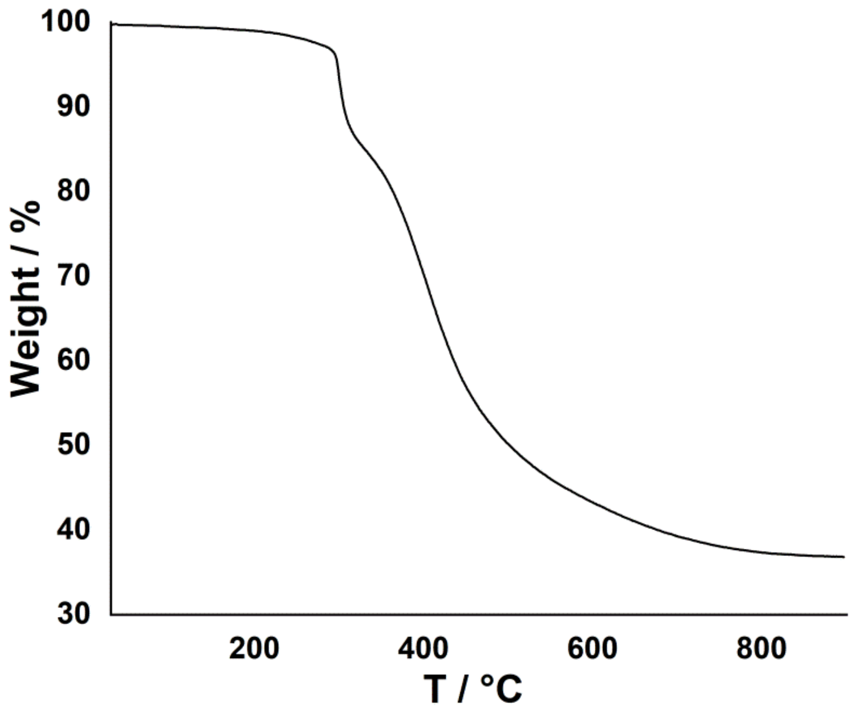

Supplementary Fig. 5 TGA curve of CPOC-301.

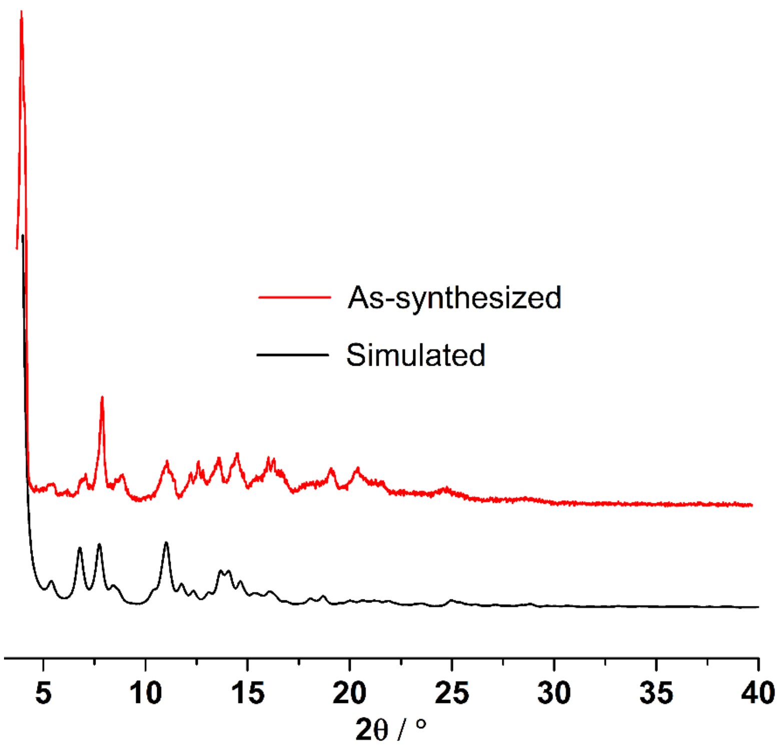

Supplementary Fig. 6 PXRD pattern of CPOC-301.

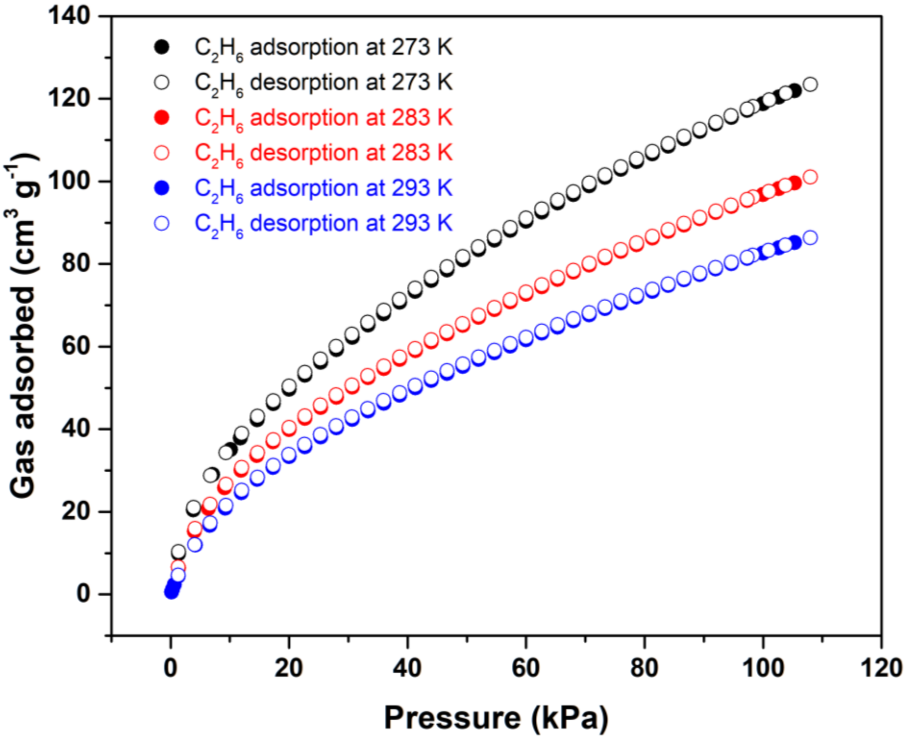

Supplementary Fig. 7 C_2H_6 adsorption/desorption isotherm of CPOC-301 at 273, 283 and 293 K.

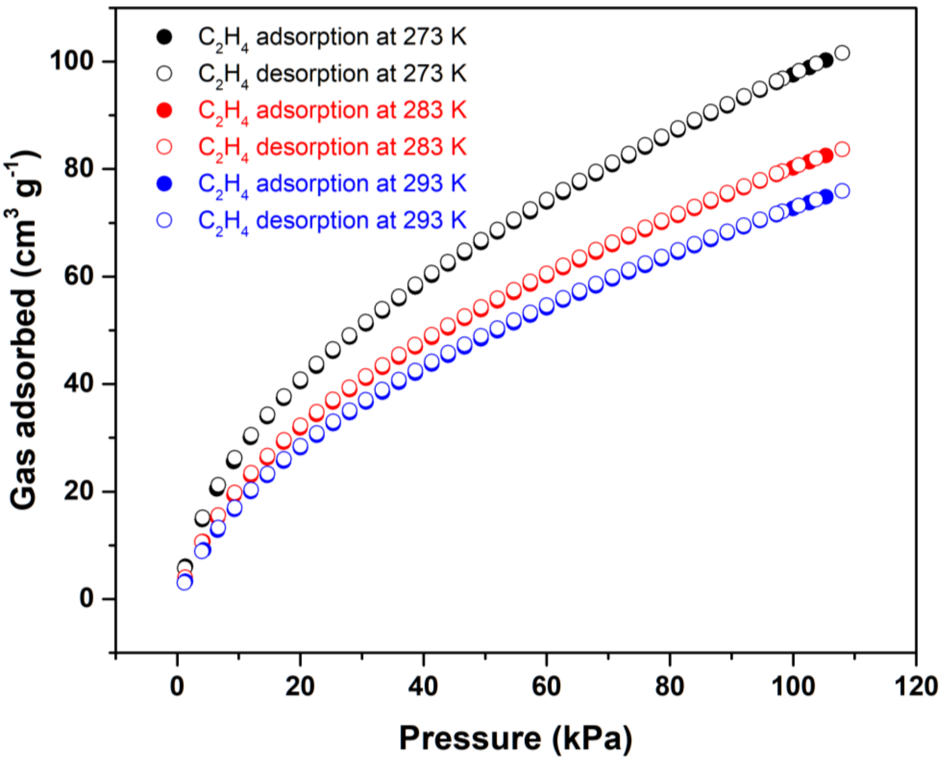

Supplementary Fig. 8 C_2H_4 adsorption/desorption isotherm of CPOC-301 at 273, 283 and 293 K.

(a)

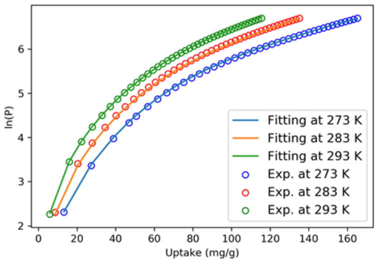

(b)

	Parameter value	Standard deviation
**a0**	-3895.385	177.8326
**a1**	23.5972	5.125331
**a2**	-5.132419E-2	4.952644E-2
**a3**	-1.312749E-3	5.581102E-4
**a4**	8.405202E-6	3.51366E-6
**a5**	-1.799377E-8	8.105978E-9
**Adj. R-Square**	0.9996	

Supplementary Fig. 9 (a) Virial equation fitting of the C_2H_6 adsorption isotherm of CPOC-301 at 273, 283 and 293 K. (b) Relevant fitting parameters for C_2H_6.

(a)

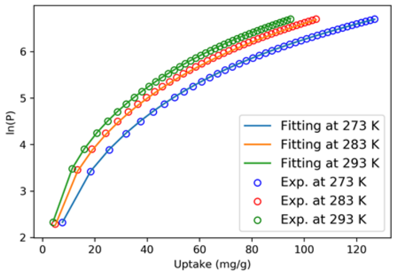

(b)

	Parameter value	Standard deviation
**a0**	-2888.351	140.9872
**a1**	13.41196	5.204910
**a2**	-1.88417E-2	6.563444E-2
**a3**	-1.055152E-3	1.000175E-3
**a4**	7.665586E-6	8.280272E-6
**a5**	-1.921508E-8	2.505110E-8
**Adj. R-Square**	0.9997	

Supplementary Fig. 10 (a) Virial equation fitting of the C_2H_4 adsorption isotherm of CPOC-301 at 273, 283 and 293 K. (b) Relevant fitting parameters for C_2H_4.

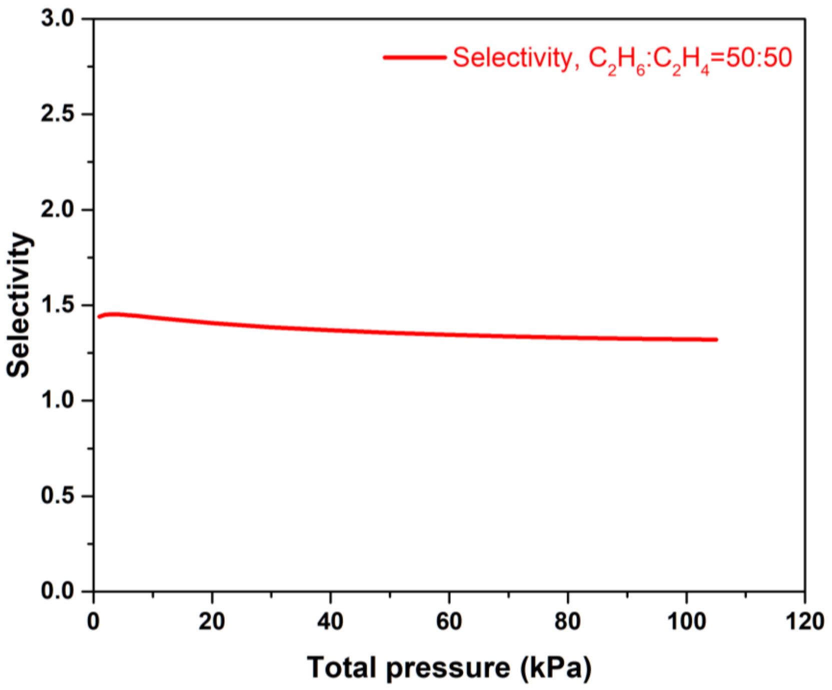

Supplementary Fig. 11 Selectivity of CPOC-301 predicted by the IAST method for an equimolar C_2H_6/C_2H_4 mixture at 293 K.

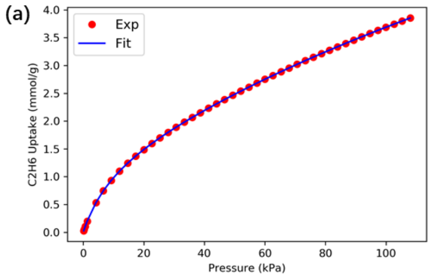

(b)

	Parameter value	Standard deviation
A1	8.201807	0.3796967
A2	1.844211	0.05399933
B1	1.080576E-3	1.026994E-4
B2	8.819491E-2	1.896082E-3
C1	1.24567	0.02814527
C2	0.967183	3.641178E-3
Adj. R-Square	0.999999	

Supplementary Fig. 12 Dual-site Langmuir-Freundlich fitting of the C_2H_6 adsorption isotherm of CPOC-301 at 293 K. (b) Relevant fitting parameters for C_2H_6.

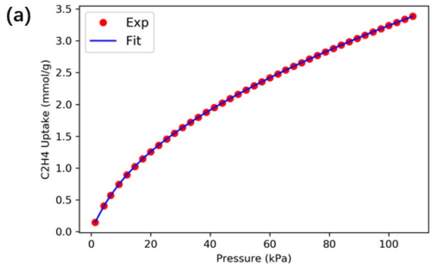

(b)

	Parameter value	Standard deviation
A1	5.518558	0.4165471
A2	2.388309	0.1088176
B1	4.205926E-4	9.145121E-5
B2	4.813541E-2	1.754617E-3
C1	1.441421	5.467777E-2
C2	0.9528982	4.886881E-3
Adj. R-Square	0.999999	

Supplementary Fig. 13 Dual-site Langmuir-Freundlich fitting of the C_2H_4 adsorption isotherm of CPOC-301 at 293 K. (b) Relevant fitting parameters for C_2H_4.

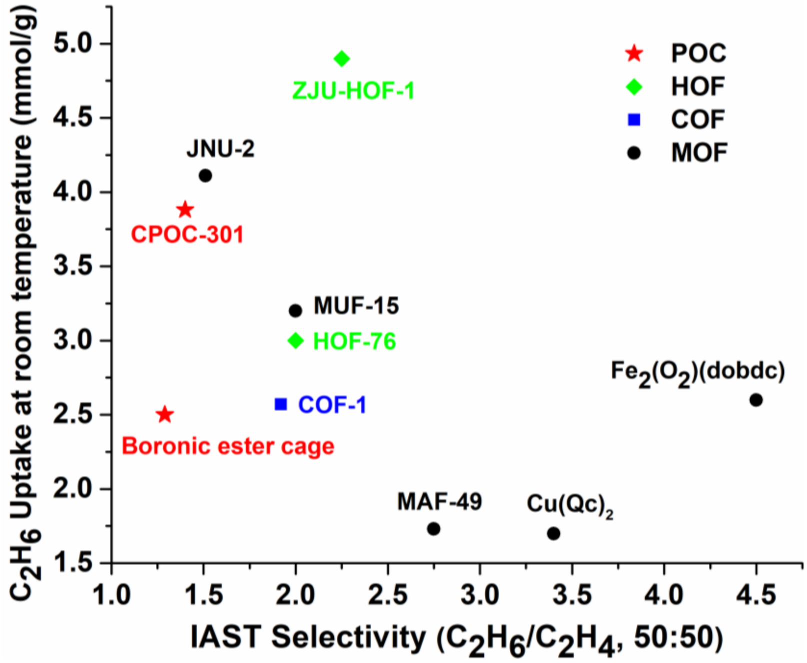

Supplementary Fig. 14 The reported C_2H_6-selective porous organic molecular materials, and several selected C_2H_6-selective porous framework materials[S1-S9]. Note: the C_2H_6 Uptake of boronic ester cage was measured at 273 K, and its actual C_2H_6/C_2H_4 separation performance by breakthrough experiment were not investigated.

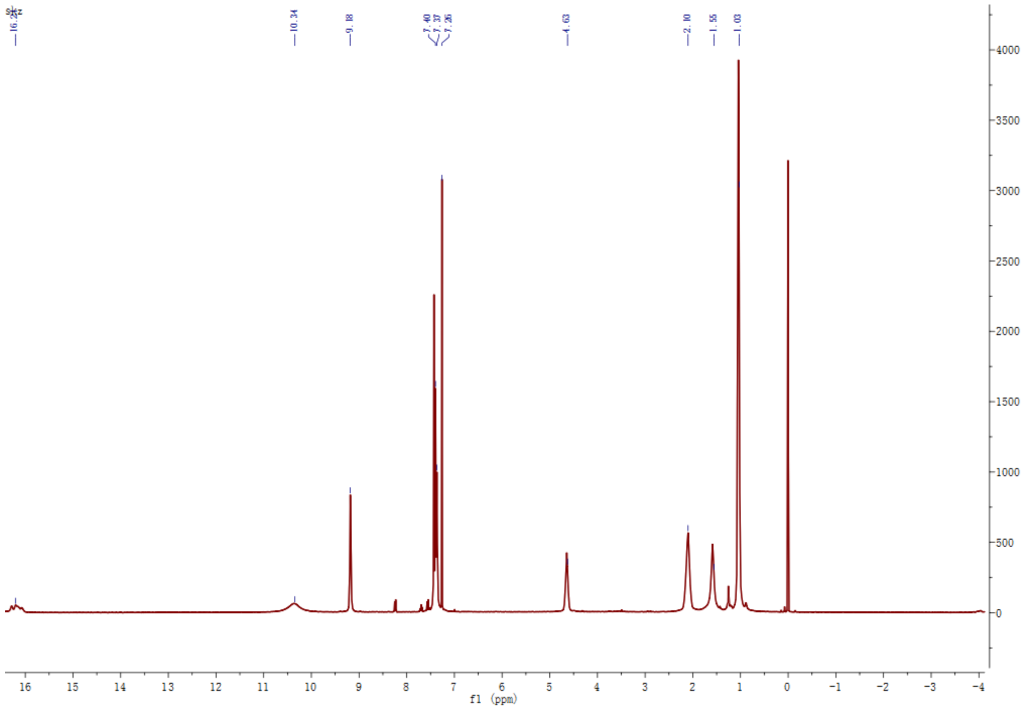

Supplementary Fig. 15 ^{1}HNMR of CPOC-301 after breakthrough experiments ($CDCl_3$, 400 MHz, 298 K).

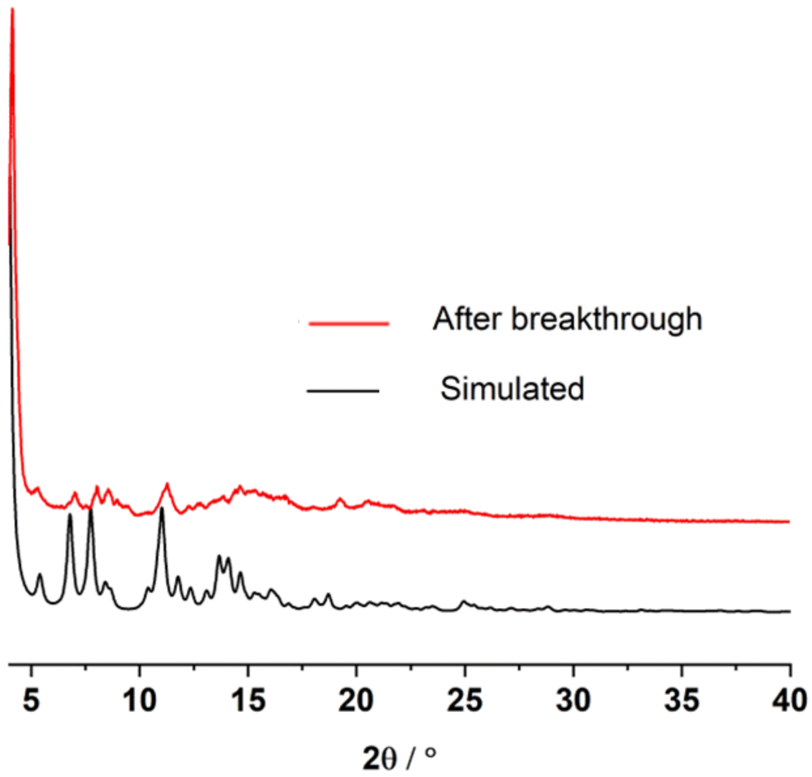

Supplementary Fig. 16 PXRD of CPOC-301 after breakthrough experiments.

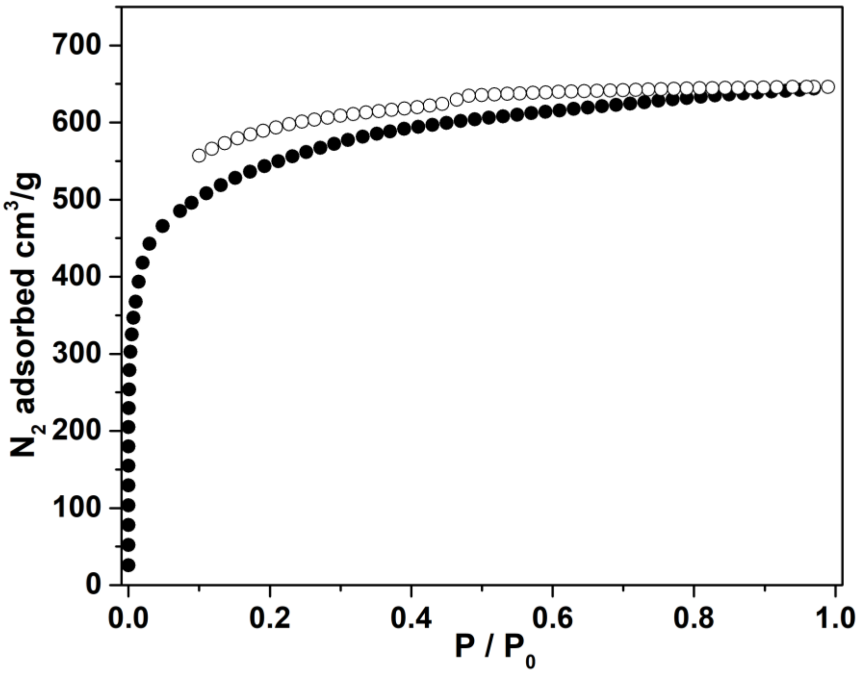

Supplementary Fig. 17 N_2 gas sorption isotherm at 77 K for CPOC-301 after being exposed to air for a week.

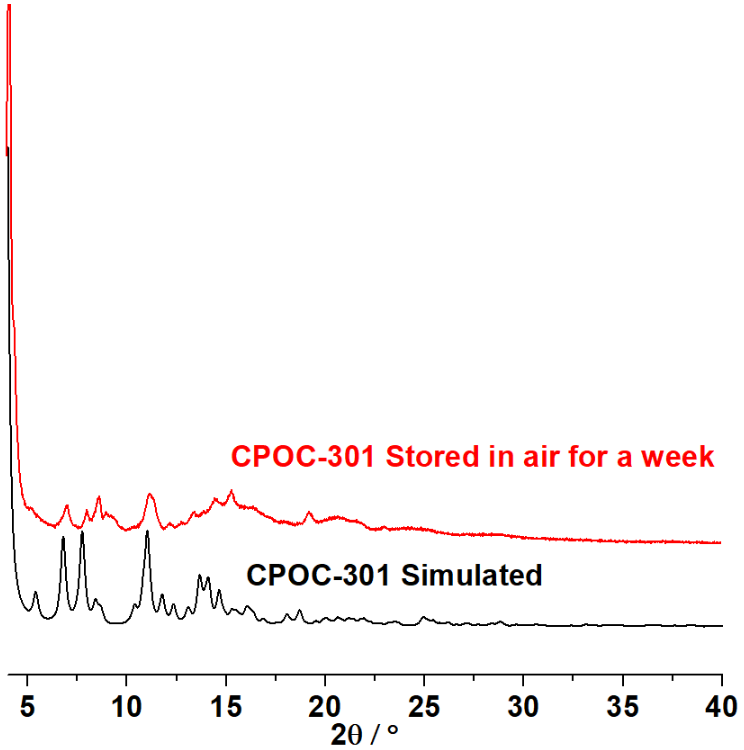

Supplementary Fig. 18 PXRD of CPOC-301 after being exposed to air for a week.

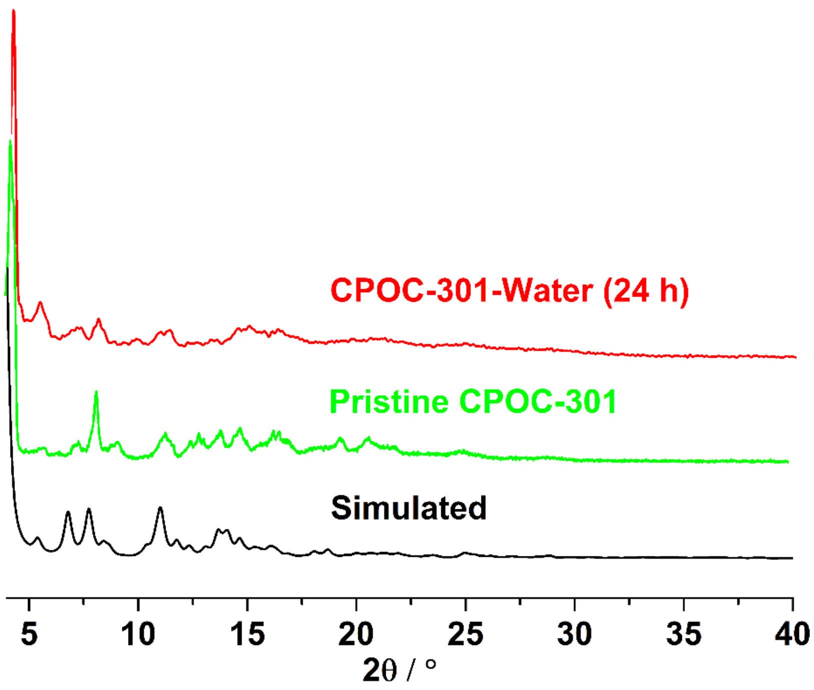

Supplementary Fig. 19 PXRD of CPOC-301 after being soaked in water for 24 hours.

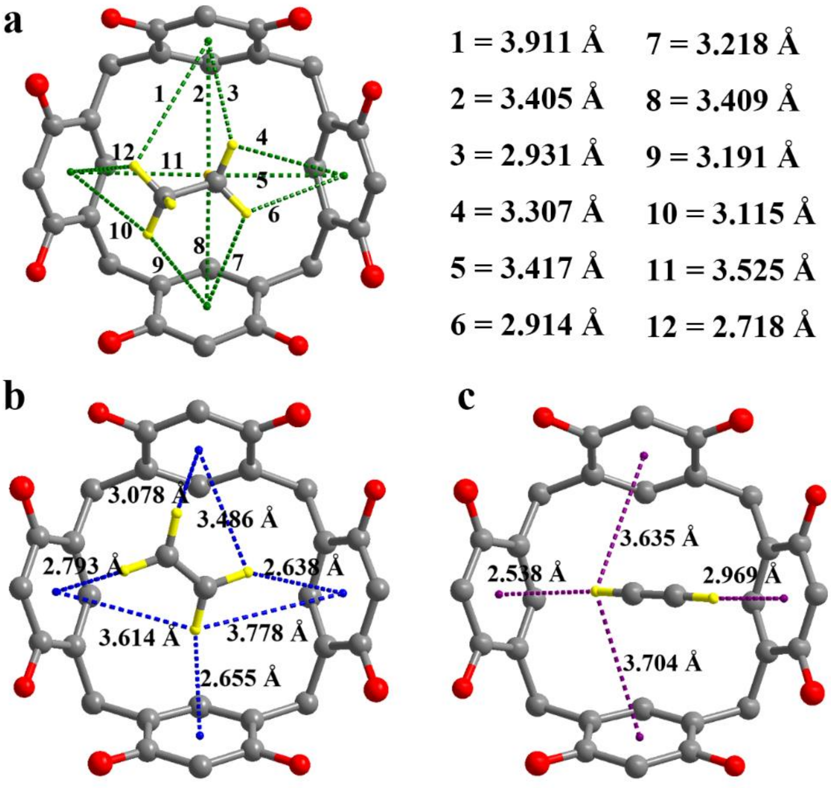

Supplementary Fig. 20 The C-H···π distances of hydrogen of **a** C_2H_6, **b** C_2H_4 and **c** C_2H_2 molecules to the center of phenyl ring of calix[4]resorcinarene.

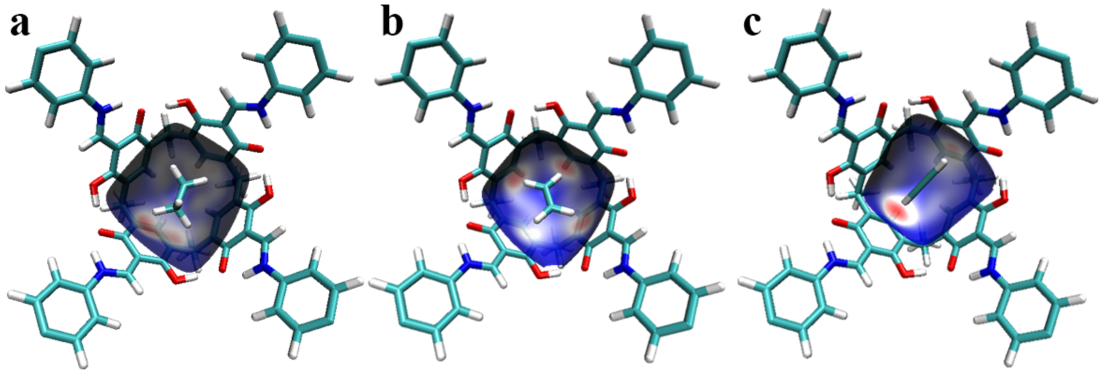

Supplementary Fig. 21 The Hirshfeld surface showing the intermolecular interactions of **a** C_2H_6, **b** C_2H_4 and **c** C_2H_2 with the cavities of calix[4]resorcinarene. Blue-white-red, which corresponds to electron density varying from 0.0 to 0.015 a.u.

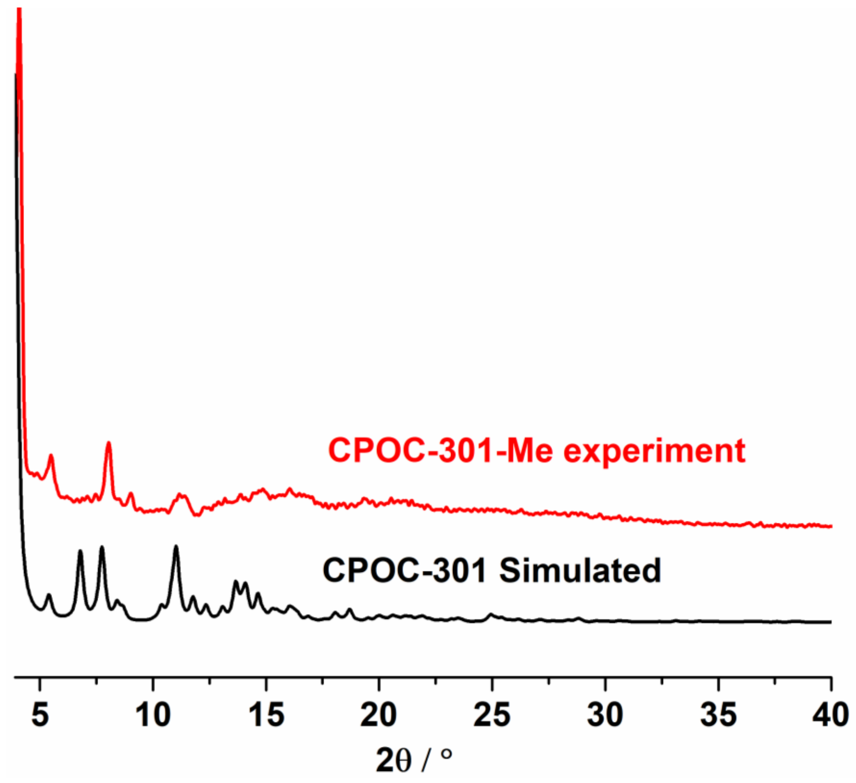

Supplementary Fig. 22 PXRD patterns of CPOC-301-Me after desolvation. This suggests that CPOC-301-Me is isostructural to CPOC-301.

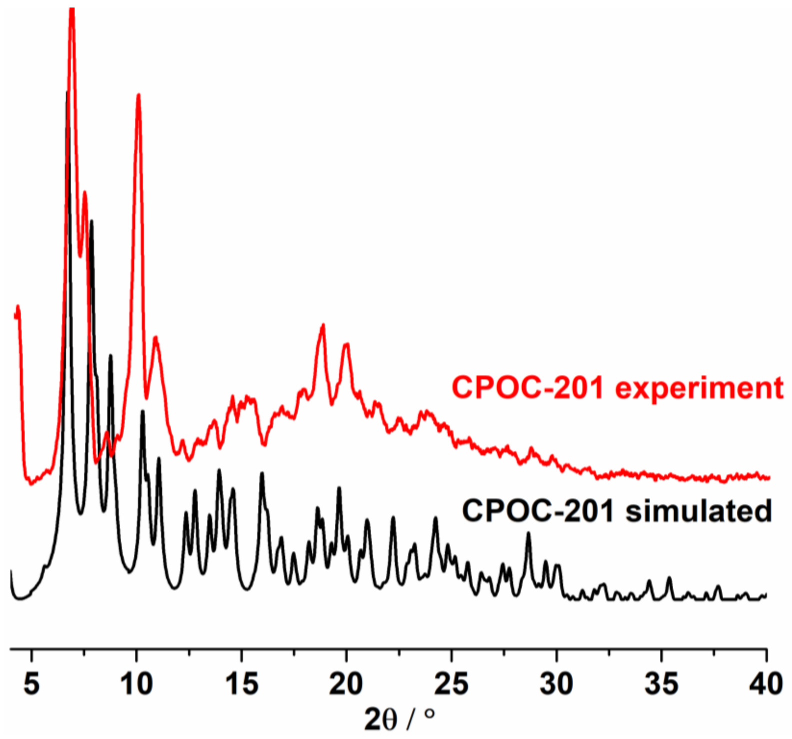

Supplementary Fig. 23 PXRD patterns of CPOC-201 after desolvation.

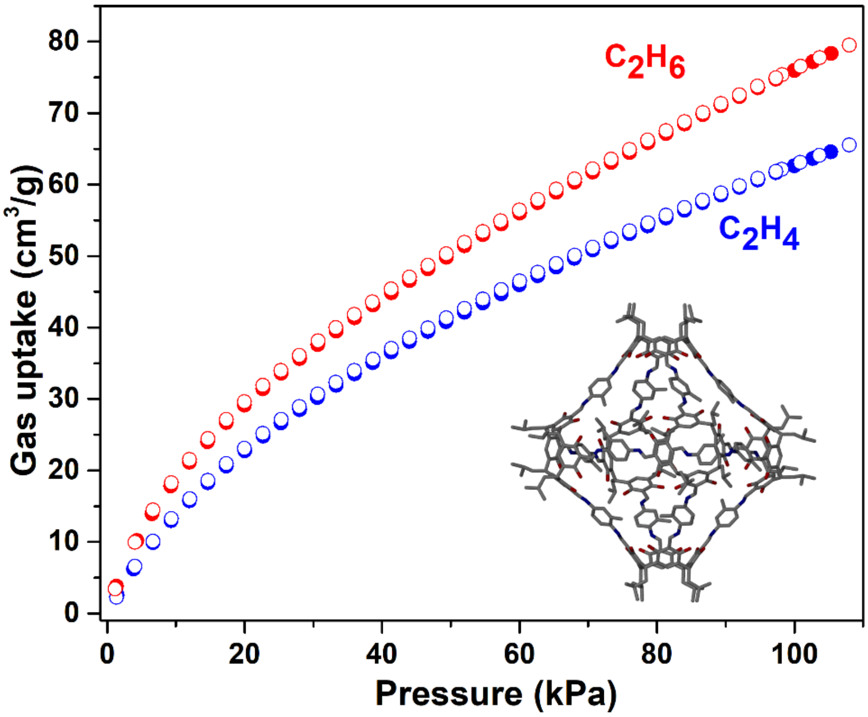

Supplementary Fig. 24 The Experimental C_2H_6 and C_2H_4 adsorption isotherms of CPOC-301-Me at 293 K. Inset is the simulated molecule structure of CPOC-301-Me.

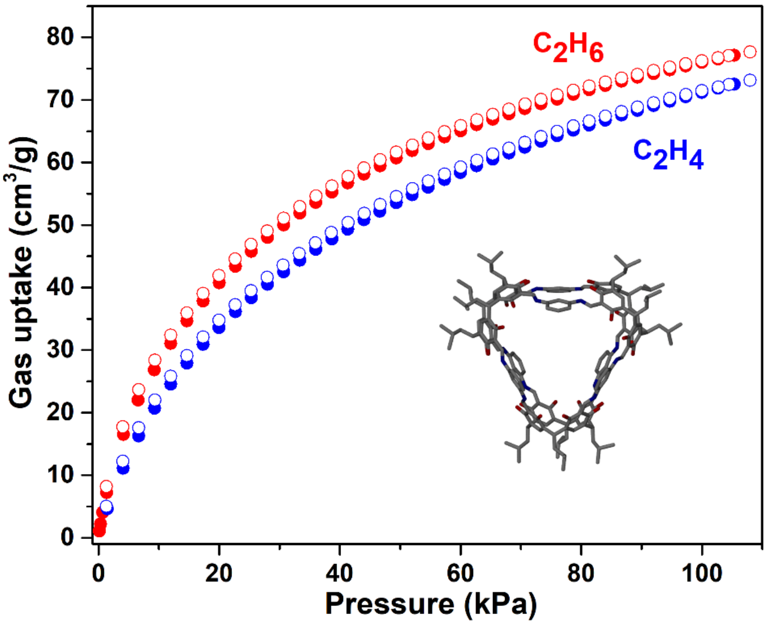

Supplementary Fig. 25 The Experimental C_2H_6 and C_2H_4 adsorption isotherms of CPOC-201 at 293 K. Inset is the molecule structure of CPOC-201.

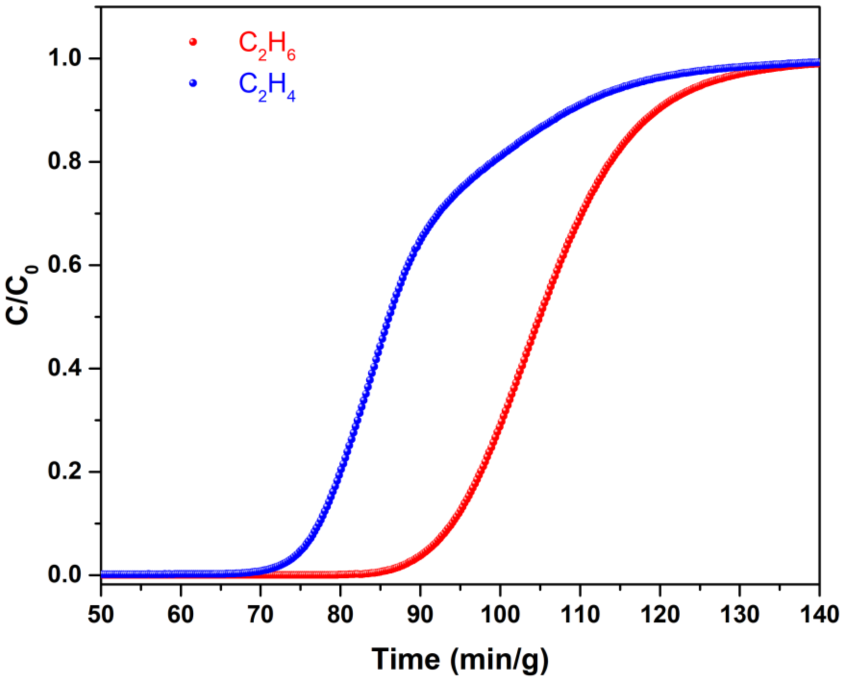

Supplementary Fig. 26 The The experimental column breakthrough curve of CPOC-301-Me with an equimolar C_2H_6/C_2H_4 mixture at 298 K. From this dynamic breakthrough experiment, the calculated separation factor for an equimolar mixture of C_2H_6/C_2H_4 was 1.18.

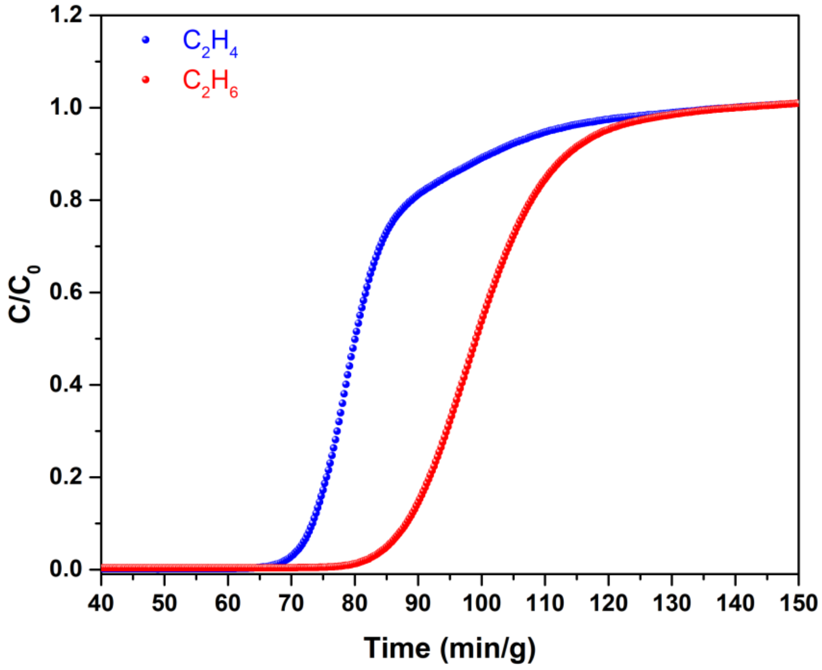

Supplementary Fig. 27 The The experimental column breakthrough curve of CPOC-201 with an equimolar C_2H_6/C_2H_4 mixture at 298 K. From this dynamic breakthrough experiment, the calculated separation factor for an equimolar mixture of C_2H_6/C_2H_4 was 1.20.

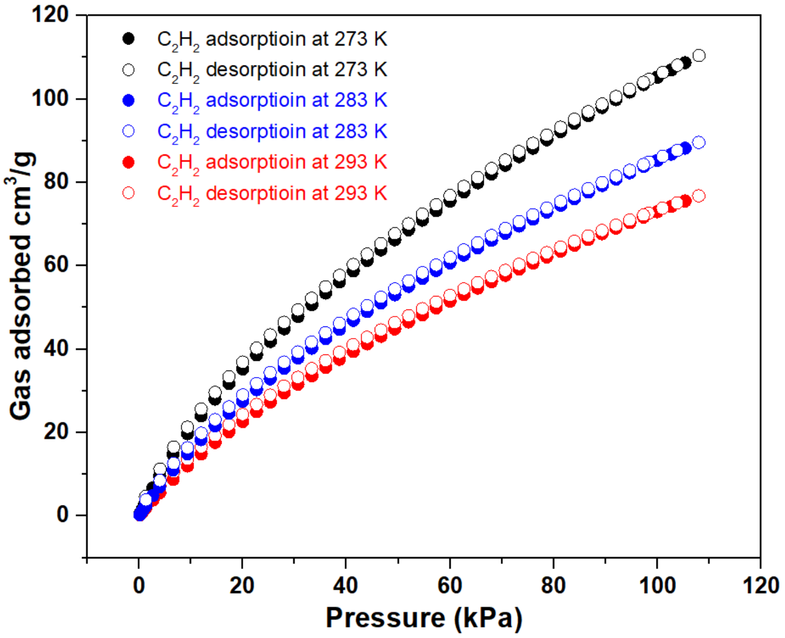

Supplementary Fig. 28 C_2H_2 adsorption/desorption isotherm of CPOC-301 at 273, 283 and 293 K.

(a)

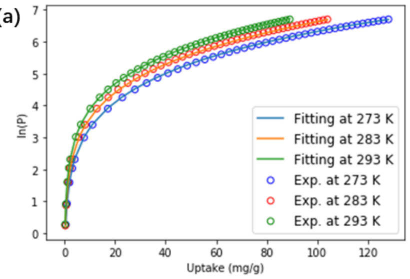

(b)

	Parameter value	Standard deviation
a0	-2431.677	64.59064
a1	7.11924	3.139478
a2	-5.18376E-2	4.08447E-2
a3	-3.821604E-4	6.343808E-4
a4	3.735269E-6	5.643884E-6
a5	-1.380009E-8	1.141135E-8
Adj. R-Square	0.9999	

Supplementary Fig. 29 (a) Virial equation fitting of the C_2H_2 adsorption isotherm of CPOC-301 at 273, 283 and 293 K. (b) Relevant fitting parameters for C_2H_2.

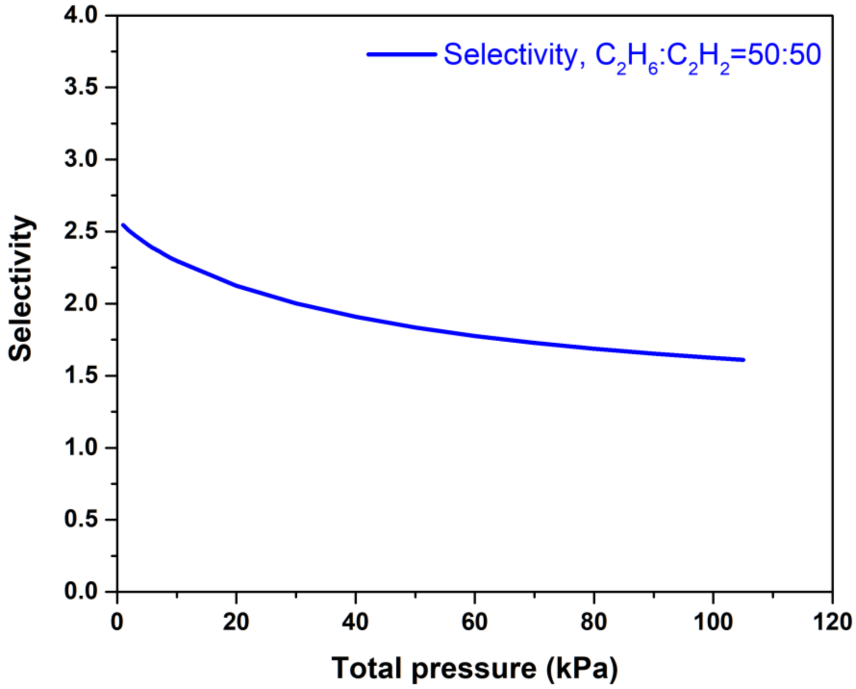

Supplementary Fig. 30 Selectivity of CPOC-301 predicted by the IAST method for an equimolar C_2H_6/C_2H_2 mixture at 293 K.

(a)

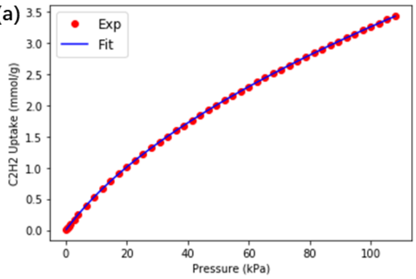

(b)

	Parameter value	Standard deviation
A1	7.104384	1.219073
A2	2.745464	0.3596393
B1	4.118428E-04	1.989202E-04
B2	2.37587E-02	2.388391E-03
C1	1.389373	0.1147201
C2	0.9685324	3.769259E-03
Adj. R-Square	0.999999	

Supplementary Fig. 31 Dual-site Langmuir-Freundlich fitting of the C_2H_2 adsorption isotherm of CPOC-301 at 293 K. (b) Relevant fitting parameters for C_2H_2.

Supplementary Table 1 The binding energies (ΔE) for C2@CPOC-301 calculated by the Dmol3 module.

	$E_{POC+gas}$ (ha)	E_{POC} (ha)	E_{gas} (ha)	ΔE (ha)	ΔE (kJ/mol)
C_2H_6	-16112.1123535	-16032.3607175	-79.7353730	-0.0162630	-42.7
C_2H_4	-16110.8772222	-16032.3607175	-78.5008139	-0.0156908	-41.2
C_2H_2	-16109.6227364	-16032.3607175	-77.2480486	-0.0139703	-36.7

References

S1. Elbert S. M., *et al.* Shape-persistent tetrahedral 4+6 boronic ester cages with different degrees of fluoride substitution. *Chem. Eur. J.* **24**, 11438-11443 (2018).

S2. Zhang X., *et al.* Selective ethane/ethylene separation in a robust microporous hydrogen-bonded organic framework. *J. Am. Chem. Soc.* **142**, 633-640 (2020).

S3. Chen B., *et al.* A rod-packing hydrogen-bonded organic framework with suitable pore confinement for benchmark ethane/ethylene separation. *Angew. Chem. Int. Ed.* **60**, 10304-10310 (2021).

S4. He C. H., *et al.* Microregulation of pore channels in covalent-organic frameworks used for the selective and efficient separation of ethane. *ACS Appl. Mat. Interfaces.* **12**, 52819-52825 (2020).

S5. Li L., *et al.* Ethane/ethylene separation in a metal-organic framework with iron-peroxo sites. *Science.* **362**, 443-446 (2018).

S6. Qazvini O. T., Babarao R., Shi Z.-L., Zhang Y.-B. & Telfer S. G. A robust ethane-trapping metal-organic framework with a high capacity for ethylene purification. *J. Am. Chem. Soc.* **141**, 5014-5020 (2019).

S7. Lin R.-B., *et al.* Boosting ethane/ethylene separation within isoreticular ultramicroporous metal-organic frameworks. *J. Am. Chem. Soc.* **140**, 12940-12946 (2018).

S8. Zeng H., *et al.* Cage-interconnected metal-organic framework with tailored apertures for efficient C_2H_6/C_2H_4 separation under humid conditions. *J. Am. Chem. Soc.* **141**, 20390-20396 (2019).

S9. Liao P.-Q., Zhang W.-X., Zhang J.-P. & Chen X.-M. Efficient purification of ethene by an ethane-trapping metal-organic framework. *Nat. Commun.* **6**, 9697 (2015).
